# Forehead Regional Oxygen Saturation (rSO2)-Related Ear-Level Arterial Pressure and Lower Thigh rSO2 in the Steep Trendelenburg Position with CO2 Pneumoperitoneum and the Beach Chair Position

**DOI:** 10.7759/cureus.15687

**Published:** 2021-06-16

**Authors:** Tomoko Fukada, Yuri Tsuchiya, Hiroko Iwakiri, Makoto Ozaki, Minoru Nomura

**Affiliations:** 1 Department of Anesthesiology, Tokyo Women’s Medical University, School of Medicine, Tokyo, JPN

**Keywords:** regional oxygen saturation, forehead, lower thigh, mean arterial pressure, heart-level, ear-level

## Abstract

Introduction

Regional oxygen saturation (rSO2) reflects tissue perfusion. This observational study aimed to examine the change in the forehead and lower thigh rSO2 associated with intraoperative posture, anesthesia regimen, or mean arterial pressure (mAP) at heart and external auditory meatus (ear) levels.

Methods

Patients undergoing robot-assisted laparoscopic radical prostatectomy in the Trendelenburg position at 30° with pneumoperitoneum (TPP) or arthroscopic shoulder surgery in the beach chair position at 70° (BCP) under desflurane-remifentanil (D/R) or propofol-remifentanil (P/R) anesthesia were examined. Bilateral forehead and lower thigh rSO2 values and mean radial artery pressure were measured simultaneously at heart and ear levels.

Results

In TPP, there were no differences under anesthesia regimens in the forehead or lower thigh rSO2change, although one patient with an absolute lower thigh rSO2 of ≤50% in the lithotomy position complained of transient limb pain. No correlation was observed between rSO2 and mAP. In BCP, forehead rSO2 decreased and lower thigh rSO2 increased under either of the anesthesia regimens. The coefficient of correlation between forehead rSO2 andheart-level and ear-level mAP was 0.341 and 0.236, respectively.

Conclusions

There were no differences under anesthesia regimens in the changes of forehead rSO2 and lower thigh rSO2. In TPP, significant changes in forehead rSO2 and lower thigh rSO2 were not observed. Monitoring lower thigh rSO2_ _might be useful for preventing lower extremity pain. In BCP, forehead rSO2 decreased and lower thigh rSO2 increased from the supine position to the BCP. To prevent brain damage, anesthesiologists should pay attention to heart- and ear-level mAP.

## Introduction

Regional oxygen saturation (rSO2) reflects tissue perfusion and thus should be monitored closely during certain types of surgeries, especially cerebral rSO2. During cardiac surgery, a decrease in forehead rSO2 has been associated with cognitive dysfunction and delirium as well as prolonged hospital stay after surgery [1-3]. Similarly, frontal lobe rSO2 has been measured to predict cerebral ischemia during carotid endarterectomy and to detect postoperative stroke and cerebral hyperperfusion syndrome after carotid endarterectomy [4-6]. Certain surgical positions can exacerbate these changes in rSO2. For instance, robot-assisted laparoscopic radical prostatectomy (RALP) is performed in the Trendelenburg position at 30° with pneumoperitoneum (TPP), which affects the intracranial pressure (ICP) and thus alters cerebral blood flow (CBF) and bilateral forehead rSO2. Arthroscopic shoulder surgery in the beach chair position (BCP) may cause serious neurological injury owing to a decrease in cerebral perfusion [7,8]. Therefore, changes only in forehead rSO2 during RALP or BCP were measured [9-13]; only one study measured forehead rSO2 and lower thigh rSO2 during RALP [14].

Thus, our primary objective was to simultaneously examine the change in the forehead and lower thigh rSO2, associated with TPP or BCP. The secondary objective was to evaluate the influence of mean arterial pressure (mAP) at the heart level and the external auditory meatus (ear) level on forehead rSO2 change.

The change in rSO2 during RALP and arthroscopic shoulder surgery as shown in this study were presented as a poster at the International Society for Anaesthetic Pharmacology 26th Annual Meeting, on October 20, 2017.

## Materials and methods

Patients

Our study was approved by the institutional review board of Tokyo Women’s Medical University (3945, 3945-R1, and 3945-R2; approval date May 28, 2016; July 25, 2017; and March 22, 2019) and registered at University Hospital Medical Information Network Clinical Trials Registry (UMIN-CTR) on July 13, 2016 (UMIN000023155). Intravenous anesthetics were added in approval number 3945-R1, and the study period was extended in approval number 3945-R2. Seventy-nine patients aged ≥ 20 years undergoing RALP in TPP or arthroscopic shoulder surgery in BCP were enrolled from July 22, 2016, to November 25, 2019. The exclusion criteria were a history of cerebrovascular diseases or stenosis of the neck vessels. After we explained the study procedures and goals, we obtained written informed consent from all the patients. 

rSO2 measurement

Four-channel rSO2 was measured using the INVOS™ 5100C Cerebral/Somatic Oximetry system and INVOS™ 5100C Cerebral/Somatic Oximetry Adult Sensors (Medtronic, Minneapolis, Minnesota, USA). Before the induction of anesthesia, rSO2 sensors were affixed to the bilateral forehead and the lower thighs. The rSO2 levels of the bilateral forehead and lower thighs were measured during spontaneous breathing before anesthesia induction in the supine (baseline) position; after intubation; 0 (immediately), 5, 10, 20, 30, 60, 90, 120, and 150 minutes after changing position; immediately and 10 minutes after returning to the supine position; and before discharge from the operating room. We evaluated the absolute rSO2 value at each measurement point and the number of cases in which rSO2 decreased to ≤80% of baseline or in which the absolute rSO2 decreased to <50% in more than 5 minutes after a position change. The rSO2 values were only known to the data collector (AZ), who alerted the anesthesiologist when the absolute lower thigh rSO2 decreased to ≤50% during RALP. 

Anesthetic management

Bispectral index (BIS; using the BIS A-2000 [Nihon Kohden, Tokyo, Japan]), noninvasive blood pressure of the arm, heart rate (HR), and peripheral oxygen saturation (SpO2) using the bedside monitor DS-8500 (Fukuda Denshi, Tokyo, Japan) were monitored simultaneously. Briefly, after induction of anesthesia, a 22G arterial catheter (BD Insyte-ATM, Nippon Becton Dickinson, Tokyo, Japan) was inserted percutaneously into the radial artery, and mAP was measured by positioned transducers at 5 cm below the sternal angle (heart level) and the external auditory meatus height (ear level).

Patients undergoing RALP were placed in the lithotomy position using Levitator© (Mizuho, Tokyo, Japan) and in TPP after the insertion of medical instruments into their abdomen for prostatectomy. Intra-abdominal pressure was maintained at <15 mmHg. Patients undergoing arthroscopic shoulder surgery patients were placed in BCP at 70° using the beach chair shoulder positioning system (Conmed, Utica, New York, USA), after tracheal intubation until the end of the operation. Arthroscopic shoulder surgery was performed under general anesthesia with interscalene brachial plexus block using levobupivacaine or ropivacaine, except in four cases, wherein patients did not accept regional anesthesia. The dose and timing of intravenous fentanyl administration during the operation and strategies for postoperative analgesia were at the discretion of the anesthesiologist.

Ten patients undergoing RALP and 10 undergoing arthroscopic shoulder surgery were anesthetized with desflurane-remifentanil (D/R) according to approval number 3945. The remaining patients undergoing each procedure were anesthetized with D/R or propofol-remifentanil (P/R) anesthesia according to approval number 3945-R1. The administration of D/R or P/R was at the discretion of the anesthesiologists. None of the patients were premedicated. In the D/R anesthesia group, general anesthesia was induced using intravenous 1−2 mg/kg propofol, followed by 0.1 mg fentanyl, 0.5 μg/kg/min remifentanil, and 0.6 mg/kg rocuronium for intubation. Anesthesia was maintained at a BIS of 40−60 with desflurane (<0.7 minimum alveolar concentration) and 0.2−0.5 μg/kg/min remifentanil. In the P/R anesthesia group, anesthesia was induced with 4 μg/mL propofol using target-controlled infusion (TCI) and maintained at a BIS of 40−60 with TCI (Marsh model) using the Terfusion® TCI pump CoTE-371 (Terumo, Tokyo, Japan) and 0.2−0.5 μg/kg/min remifentanil. Rocuronium was administrated at 7 μg/kg/min from intubation to the end of surgery in both anesthesia groups. The ventilator settings were as follows: tidal volume 8 mL/kg, inspiratory: expiratory ratio 1:2, inspired O2 fraction (FiO2) 45% with air, and positive end-expiratory pressure 5 cmH2O. The respiratory rate was adjusted to maintain end-tidal CO2 (ETCO2) at 35−40 mmHg. The rate of intravenous crystalloid infusion was kept constant at approximately 8 mL/kg/h. After anesthesia induction, all patients wore surgical stockings and a foot pump (Kendall SCDTM 700, CardinalHealth, Ohio, USA) to prevent deep vein thrombosis. For patients whose pharyngeal or bladder temperature decreased to <36.5 °C, a warmer blanket was used to prevent hypothermia. Patients who met discharge criteria with an Aldrete score of ≥9 were moved to their respective wards. Postoperative cognitive dysfunction and delirium were checked through postoperative rounds and medical records.

During surgery, systolic arterial pressure (sAP) of ≤80 mmHg at heart level was treated with ephedrine 4−8 mg or phenylephrine 0.05−0.1 mg, whereas sAP of ≥160 mmHg at heart level was treated with nicardipine 0.5 mg. The administration of ephedrine or phenylephrine was at the discretion of anesthesiologists. The patients who were administered other cardiovascular agents were excluded because the interaction between these agents and anesthetics complicated the CBF and ICP changes [15]. 

Statistical analysis

The calculation of sample size was based on previous studies reporting that forehead rSO2 increased by a mean of 3% (standard deviation [SD] = 7%) or decreased by a mean of 4% (SD = 7%) after shifting from the supine position to RALP [9, 10, 14] or BCP to maintained anesthesia using intravenous anesthetics or inhalation anesthetics [8, 12, 16]. For a power of 0.8 and a P-value of 0.05, a sample size of 65 patients was calculated to enable the detection of a clinically relevant change in rSO2. All data are expressed as mean (SD). In each position, differences due to anesthesia regimen were compared using Pearson’s chi-square test or Student’s unpaired t-test. A repeated multivariate analysis of variance was performed to identify the association of anesthesia regimen with the time of change of rSO2. Changes from baseline were assessed by Dunnett’s test. For all tests, P <0.05 (two-tailed) was considered statistically significant. We calculated Pearson's correlation coefficient between rSO2 and mAP.

## Results

**Figure 1:**
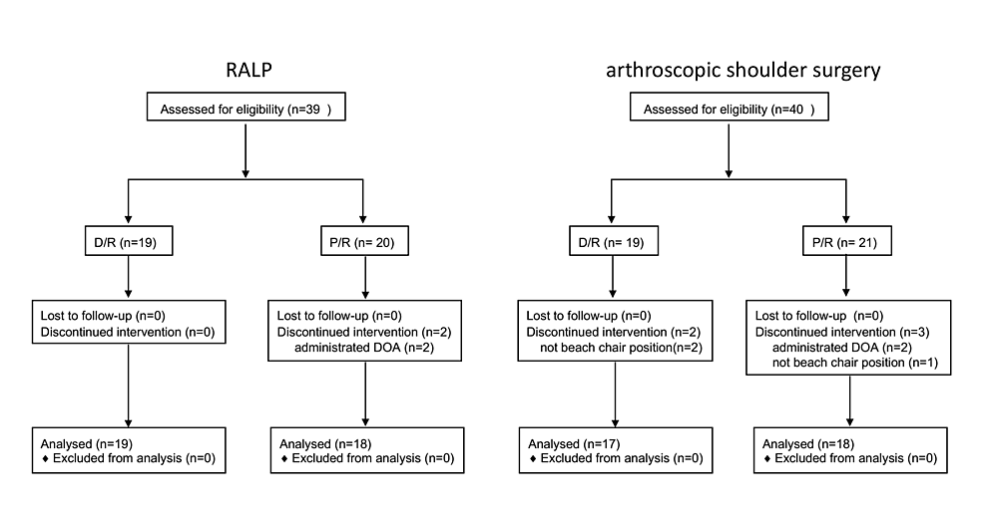
Graphical depiction of subject flow through the study. RALP: robot-assisted laparoscopic radical prostatectomy, D/R: desflurane-remifentanil, P/R: propofol-remifentanil, DOA: dopamine According to approval number 3945, patients were anesthetized by D/R. After approval number 3945-R1, patients were anesthetized by D/R or P/R, which was decided by the anesthesiologists.

Thirty-nine patients receiving RALP were assessed for eligibility; of these, two were excluded because of dopamine administration during surgery. Forty patients undergoing arthroscopic shoulder surgery patients were assessed for eligibility; among these, four were excluded because they were administered dopamine during surgery and three were not operated in BCP (Figure 1).

Data were collected on 37 RALP patients in TPP (19 D/R anesthesia vs. 18 P/R anesthesia) and 35 arthroscopic shoulder surgery patients in BCP (17 D/R anesthesia vs. 18 P/R anesthesia). Forehead rSO2 was not compared with lower thigh rSO2 in either position because they were different organs. There were no bilateral differences in the forehead or lower thigh rSO2 under any condition, so we compared the average rSO2 values at the forehead and the lower thighs. Table 1 and Table 2 summarize the patients’ characteristics, baseline data, and intraoperative clinical data. There were no significant differences in the baseline forehead and lower thigh rSO2 among all patients.

**Table 1:** Patient characteristics, baseline data, and intraoperative clinical data in TPP. Data are presented as mean (SD);  *P <0.05 by Student’s unpaired test; **P <0.05 by Fisher’s exact test TPP: steep Trendelenburg position with pneumoperitoneum, D/R: desflurane-remifentanil, P/R: propofol-remifentanil, ASA PS: American Society of Anesthesia physical status, HTN: hypertension, DM: diabetes malleus, COPD: chronic obstructive pulmonary disease, TCI: target-controlled infusion, rSO2: regional oxygen saturation, mAP: mean blood pressure, HR: heart rate

	D/R (n=19)	P/R (n=18)	P-value
Age (years)	69 (5)	65 (8)	0.070
Sex (M/F)	19/0	18/0	-
Height (cm)	168 (7)	167 (6)	0.676
Weight (kg)	69 (8)	69 (9)	0.925
ASA PS (I/II/III)	3/14/2	1/15/2	0.604
Preoperative hemoglobin (g/dL)	14.3 (1.2)	14.1 (1.4)	0.631
Comorbidities (HTN, DM, COPD)	9	11	0.402
Baseline rSO2 (%) forehead	70 (7)	72 (7)	0.252
Baseline rSO2 (%) lower thigh	66 (9)	68 (10)	0.916
Baseline mAP (mmHg)	110 (14)	115 (16)	0.282
Baseline HR (bpm)	73 (13)	76 12)	0.494
Duration of anesthesia (min)	296(52)	350 (103)	0.048*
Duration of operation (min)	220 (53)	261 (109)	0.150
Duration of TPP (min)	205 (54)	243 (83)	0.129
Volume of infusion (mL)	1936 (378)	2121 (552)	0.267
Volume of bleeding (mL)	72 (43)	68 (101)	0.863
Volume of urine (mL)	238 (217)	317 (391)	0.459
Total dose of remifentanil (μg/kg/min)	0.31 (0.09)	0.32 (0.06)	0.567
Total dose of fentanyl (μg/kg/min)	0.03 (0.01)	0.03 (0.01)	0.859
Total dose of ephedrine (μg/kg/min)	0.28 (0.3)	0.47 (0.40)	0.895
Total dose of phenylephrine (μg/kg/min)	0.00 (0.00)	0.01 (0.01)	0.030
No of patients administrated ephedrine	12	14	0.476
No of patients administrated phenylephrine	3	10	0.049**

**Table 2:** Patient characteristics, baseline data, and intraoperative clinical data in BCP. Data are presented as mean (SD);  *P <0.05 by Student’s unpaired test; **P <0.05 by Fisher’s exact test BCP: beach chair position, D/R: desflurane-remifentanil, P/R: propofol-remifentanil, ASA PS: American society of anesthesia physical status, HTN: hypertension, DM: diabetes malleus, COPD: chronic obstructive pulmonary disease, TCI: target-controlled infusion, rSO2: regional oxygen saturation, mAP: mean arterial pressure, HR: heart rate

	D/R (n=17)	P/R (n=18)	P-value
Age (years)	58 (13)	55 (14)	0.538
Sex (M/F)	9/8	7/11	0.404
Height (cm)	162 (11)	163 (12)	0.684
Weight (kg)	61 (14)	66 (14)	0.247
ASA PS (I/II/III)	7/9/1	6/12/0	0.478
Preoperative hemoglobin (g/dL)	14.0 (1.5)	13.7 (1.４)	0.537
Comorbidities (HTN, DM, COPD)	2	6	0.637
Baseline rSO2 (%) forehead	66 (11)	70 (7)	0.833
Baseline rSO2 (%) lower thigh	67 (8)	73 (11)	0.412
Baseline mAP (mmHg)	105 (13)	102 (11)	0.480
Baseline HR (bpm)	68 (11)	69 (11)	0.749
Duration of anesthesia (min)	207 (48)	223 (45)	0.332
Duration of operation (min)	120 (40)	119 (49)	0.935
Duration of BCP (min)	152 (45)	151 (48)	0.993
Volume of infusion (mL)	1287 (409)	1519 (416)	0.106
Volume of bleeding (mL)	6 (7)	5 (5)	0.678
Volume of urine (mL)	202 (331)	382 (506)	0.112
Total dose of remifentanil (μg/kg/min)	0.27 (0.20)	0.22 (0.09)	0.332
Total dose of fentanyl (μg/kg/min)	0.03 (0.02)	0.03 (0.01)	0/388
Total dose of ephedrine (μg/kg/min)	1.22 (1.24)	0.63 (0.45)	0.068
Total dose of phenylephrine (μg/kg/min)	0.01 (0.02)	0.03 (0.04)	0.028*
No of patients administrated ephedrine	13	15	0.612
No of patients administrated phenylephrine	7	12	0.407

**Figure 2:**
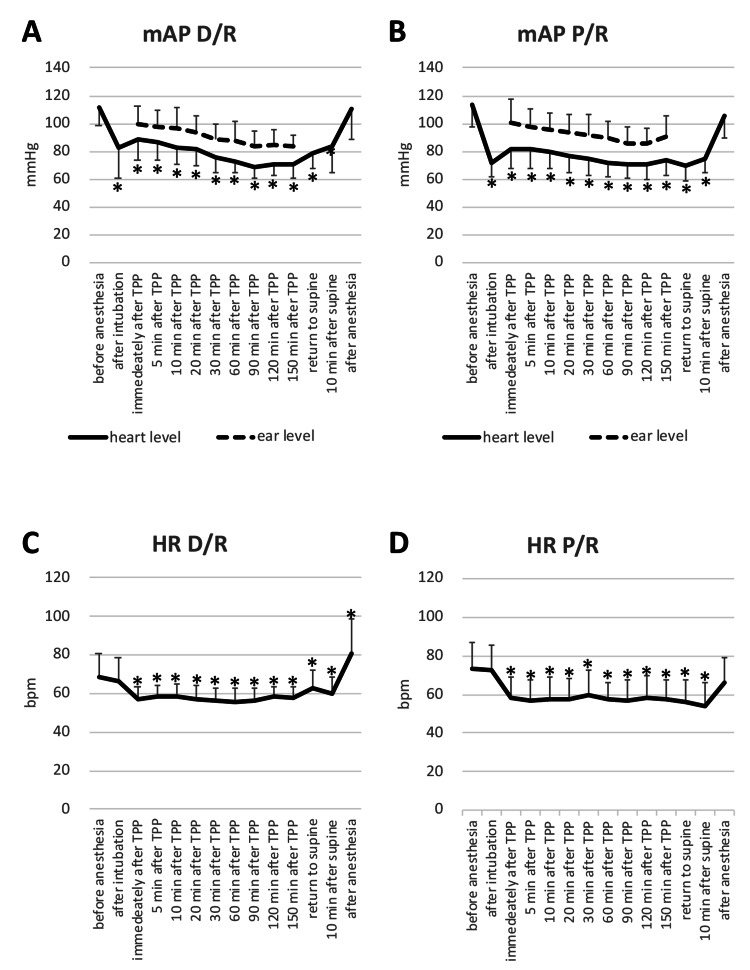
Changes in mean arterial pressure and heart rate during RALP. Data represented by mean (error bar: SD); *Compared with baseline values, P <0.05 Figure 2A, 2B: Changes in mAP at the heart and ear levels under D/R or P/R, Figure 2C, 2D: Changes in HR under D/R or P/R RALP: robot-assisted laparoscopic radical prostatectomy, mAP: mean arterial pressure, heart-level mAP: mAP measured at 5 cm under the sternal angle, ear-level mAP: mAP measured at external auditory meatus height, HR: heart rate, D/R: desflurane-remifentanil, P/R: propofol-remifentanil There were no differences in mAP and HR between the anesthetic regimens.

**Figure 3:**
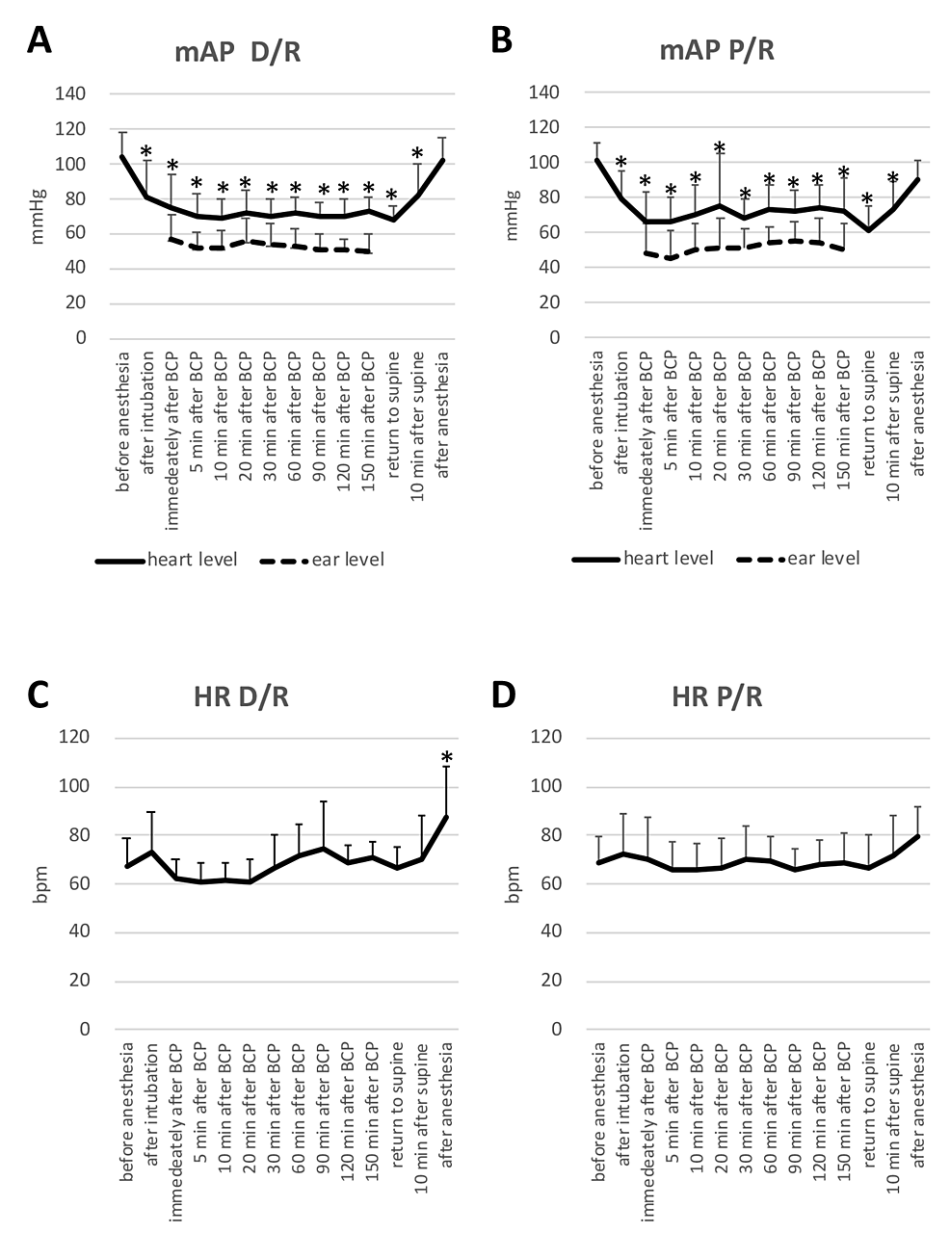
Changes in mean arterial pressure and heart rate during arthroscopic shoulder surgery. Data represented by mean (error bar: SD), *Compared with baseline values, P <0.05 Figure 3A, 3B: Changes in mAP at the heart and ear levels under D/R or P/R, Figure 3C, 3D: Changes in HR under D/R or P/R mAP: mean arterial pressure, heart-level mAP: mAP measured at 5 cm under the sternal angle, ear-level mAP: mAP measured at external auditory meatus height, HR: heart rate, D/R: desflurane-remifentanil, P/R: propofol-remifentanil There were no differences in mAP and HR between anesthetic regimens.

Figure 2 and Figure 3 present the changes in mAP at the heart and ear levels and heart rate (HR) during RALP in TPP, and during arthroscopic surgery in BCP.

Ear-level transducers were 19 (5) cm lower than heart-level transducers in TPP and 22 (5) cm higher in BCP. As compared with heart-level mAP, ear-level mAP was 16 (4) mmHg higher in TPP and 19 (6) mmHg lower in BCP. Significant differences in mAP and HR were observed between elapsed time but not anesthesia regimens during each procedure. SpO2 was ≥95% in all cases.

The pharyngeal temperature of most patients was maintained at 35.8−36.5 °C in TPP, although it was <35.0 °C in three patients. In BCP, the bladder temperature of all patients was maintained at 36.2−36.8 °C.

Change in rSO2 during RALP in TPP

**Figure 4:**
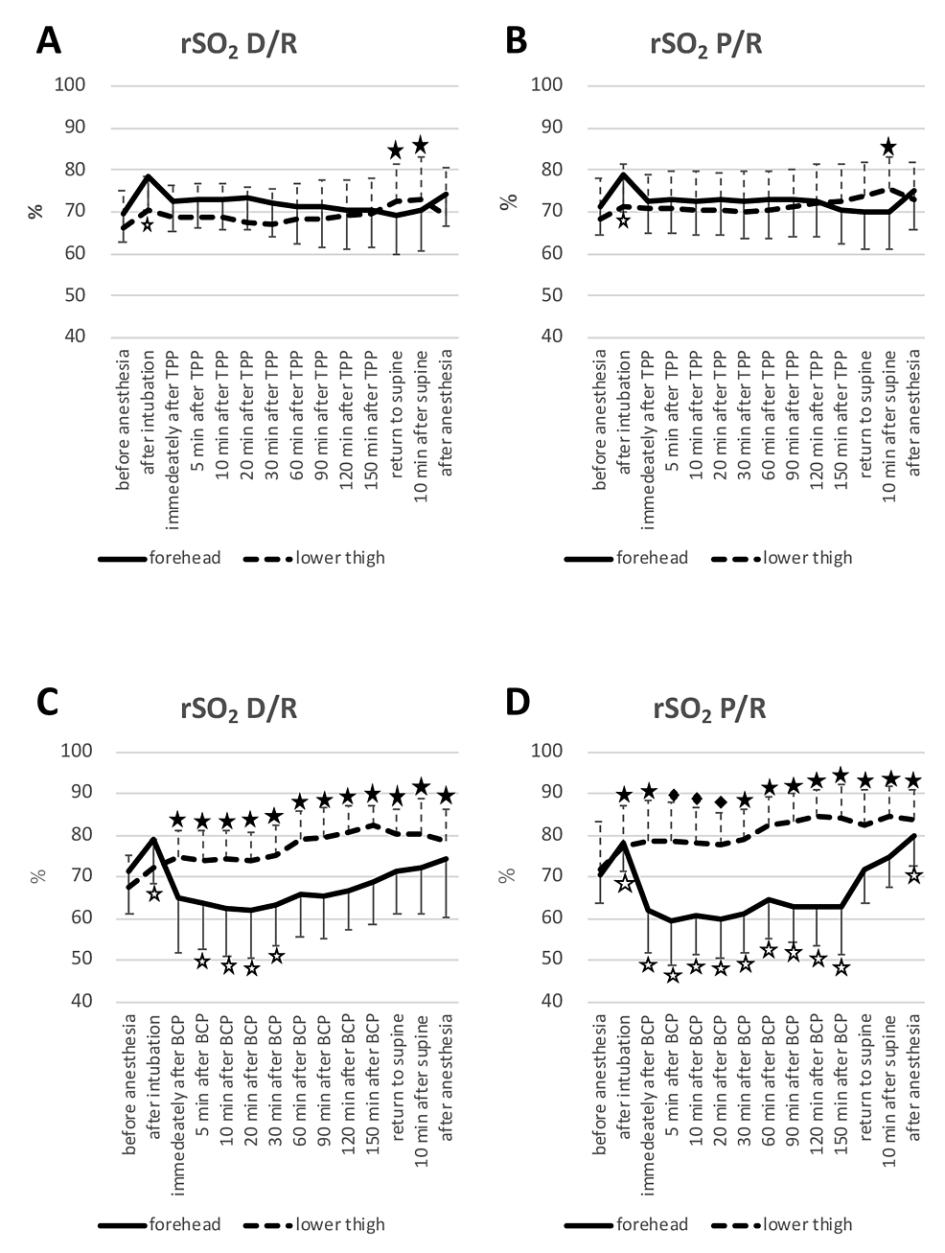
Changes in regional oxygen saturation during RALP and arthroscopic shoulder surgery. Data represented by mean (error bar: SD) Figures 4A, 4B: Changes in rSO2 under D/R or P/R in TPP, Figures 4C, 4D: Changes in rSO2 under D/R or P/R in BCP rSO2: regional oxygen saturation, RALP: robot-assisted laparoscopic radical prostatectomy, TPP: steep Trendelenburg position with pneumoperitoneum, BCP: beach chair position ☆Forehead rSO2 at each time as compared with baseline rSO2, P <0.05, ★Lower thigh rSO2 as compared with baseline rSO2 at each time point, P <0.05 During RALP, the mean (SD) baseline values of the forehead and lower thigh rSO2 under D/R anesthesia were 69% (7%) and 66% (9%), respectively. The baseline values of the forehead and lower thigh rSO2 under P/R anesthesia were 71% (7%) and 69% (10%), respectively. During arthroscopic shoulder surgery, the baseline values of the forehead and lower thigh rSO2 under D/R anesthesia were 71% (10%) and 67% (8%), respectively. The baseline values of the forehead and lower thigh rSO2 under P/R anesthesia were 70% (7%) and 73% (11%), respectively.

Changes in forehead rSO2 was associated with elapsed time but not with anesthesia regimens (P <0.0001 and P = 0.443, respectively) (Figure 4A, 4B).

Likewise, changes in lower thigh rSO2 were associated with elapsed time and not with anesthesia regimens (P <0.0001 and P = 0.568, respectively). One patient exhibited a decrease in lower thigh rSO2 for one leg at ≤50% absolute value immediately after changing from the supine to lithotomy position and complained of limb pain immediately after anesthesia termination. In this case, the duration of TPP was 3 hours 21 minutes, and the intramuscular pressures of the lower limbs were not measured. His lower limb pain was relieved on the first postoperative day without fasciotomy, and potassium concentration was 3.8 mEq/L; creatine kinase was not measured. Although it is difficult to diagnose his limb pain as lower extremity compartment syndrome, on the basis of this experience, we adjusted the lower thigh/limb position after changing to TPP when the absolute lower thigh rSO2 decreased to ≤50%. In fact, in four patients, after we rechecked the dorsalis pedis pulse, foot temperature, and knee and hip joint flexion, we changed the lower thigh/limb position in the Levitator© to recover in lower thigh rSO2 to >50 %. No correlation was noted between rSO2 and mAP (Table 3).

**Table 3:** Correlation coefficients between rSO2 and mAP. TPP: steep Trendelenburg position with pneumoperitoneum, BCP: beach chair position, rSO2: regional oxygen saturation, mAP: mean blood pressure

	Forehead rSO_2_ and heart-level mAP	Forehead rSO_2_ and ear-level mAP	Lower thigh rSO_2_ and heart-level mAP	Lower thigh rSO_2_ and ear-level mAP
TPP	0.053	0.041	-0.015	0.102
BCP	0.341	0.236	-0.093	0.005

There were no cases in which the forehead rSO2 decreased to ≤80% of baseline or in which the absolute rSO2 decreased to ≤50% at more than 5 minutes after a position change. 

No patient exhibited postoperative cognitive dysfunction, delirium, or nerve damage of the lower limb after RALP, including the one patient who complained of lower limb pain immediately after anesthesia.

Change in rSO2 during arthroscopic shoulder surgery in BCP 

Forehead rSO2 decreased and lower thigh rSO2 increased from the supine position to BCP under both anesthesia regimens. Changes in forehead rSO2 was dependent on elapsed time but not on anesthesia regimens (P <0.0001 and P = 0.794, respectively) (Figure 4C, 4D). Likewise, changes in lower thigh rSO2 were associated with elapsed time and not with anesthesia regimens (P <0.0001 and P = 0.359, respectively). We noted a weak correlation between forehead rSO2 and heart mAP and ear mAP: the correlation coefficient between forehead rSO2 and heart-level mAP and between forehead rSO2 and ear-level mAP were 0.341 and 0.236, respectively (Table 3). In six patients receiving D/R anesthesia and in eight patients receiving P/R anesthesia, forehead rSO2 decreased to ≤80% of baseline at more than 5 min after a position change. The absolute rSO2 decreased to <50% within >5 minutes after a position change in two patients in the D/R anesthesia group and in five patients in the P/R anesthesia group. None of the patients receiving arthroscopic shoulder surgery showed postoperative neurological complications.

## Discussion

In clinical practice, bilateral forehead rSO2 is measured to detect brain hypoxemia or hyperperfusion [1−6]. Oxygen supply and demand determine rSO2. Hemoglobin concentration, hemoglobin saturation, local blood flow, and cardiac output (CO) are the predominant factors determining local oxygen supply, whereas body temperature, the depth of sedation, and pain are major factors determining oxygen demand. The mean preoperative hemoglobin was 14 g/dL, which underwent almost no change during surgery because of little blood loss. Hemoglobin saturation and the body temperature were maintained at ≥95% and ~36−37 °C, respectively. Furthermore, the depth of sedation as measured by BIS of 40−60, and intraoperative pain were controlled by anesthetic delivery, and analgesics, respectively. Therefore, local blood flow and CO were important factors influencing the presence or absence of rSO2 change in TPP or BCP.

Generally, in normotensive individuals constant CBF is maintained by autoregulation within an mAP range of 50 (70)−150 mmHg, and the curves of autoregulation of CBF are flat between 50 (70)−150 mmHg [17]. By contrast, this curve shifts to the right on the BP axis in patients with uncontrolled hypertension, and this curve on the BP axis varies between the normal and right shifts in patients with well-controlled hypertension [18]. During TPP, no cases were observed in which forehead rSO2 decreased to ≤80% of the baseline or in which absolute rSO2 decreased to ≤50% for >5 minutes after a position change. Thus, mAP was within the flat section of the curves of autoregulation of CBF. In other words, CBF can also be determined by cerebral perfusion pressure (CPP), according to the previous reports, in which 170 minutes or 3 hours of TPP maintained CPP [19]. Similarly, compared with the supine position after induction of anesthesia, CO was maintained in TPP [11,20]. These studies supported our result of changes in forehead rSO2 during 150 minutes in TPP. 

No significant changes in lower thigh rSO2 at baseline were observed in TPP except in one case in which rSO2 decreased immediately after the patient position was changed from supine to lithotomy position accompanied by lower limb pain immediately after anesthesia. Because we handled this case, we adjusted the lower thigh/limb position in TPP when the absolute lower thigh rSO2 decreased to ≤50%. Only one report, which measured cerebral and lower limb rSO2 simultaneously during RALP, showed that lower limb rSO2 increased [14]. Therefore, changing the lower thigh/limb position should be considered if the lower thigh rSO2 decreases. 

During BCP, the forehead rSO2 decreased and the lower thigh rSO2 increased from the supine position to the BCP under both anesthesia regimens. To prevent cerebral ischemia, maintaining CBF and CO is critical, particularly to maintain ear-level mAP at >50 mmHg or >70 mmHg [16,21]. In this study, ephedrine or phenylephrine was administered to maintain heart-level sAP at ≥80 mmHg. This protocol may help to maintain ear-level mAP at >50 mmHg, although it is impossible to maintain it at >70 mmHg in some cases. Therefore, the coefficient of correlation between forehead rSO2 and heart-level or ear-level mAP was 0.341 and 0.236, respectively. Thus, to prevent cerebral ischemia, anesthesiologists should maintain ear-level mAP at ≥70 mmHg even if monitoring only heart-level mAP is being monitored. Conversely, lower thigh rSO2 increased to about 80% by either of the anesthesia regimens, after changing to BCP because of vessel dilatation from the anesthetics.

We noted no difference in the change in forehead rSO2 by anesthesia regimen in the TPP and BCP. These results were supported by the previous studies in which the end-tidal concentration of desflurane was <4% or the TCI level of propofol of <2.5 μg/mL did not impair cerebral autoregulation and did not decrease CBF or CO [22-24]. 

There are some limitations to this study. First, we aimed to investigate the change in rSO2 in TPP and BCP, and we compared rSO2 for 150 minutes because the mean duration of BCP was 150 minutes. If we had investigated these for >150 minutes, we could have obtained different results. We observed the change in forehead rSO2 and lower thigh rSO2 at the same time; however, we did not evaluate these changes against each other because they are different organs. Moreover, we did not assess the preoperative or postoperative cognitive function using neuropsychologic tests. However, all patients met discharge criteria with an Aldrete score of ≥9, and there were no obvious cognitive impairments during postoperative rounds or noted in the medical records. The major limitation of this study is that we did not measure CO even though we believed that mAP and CO were important factors that influence rSO2.

## Conclusions

There were no differences under anesthesia regimens in the changes of forehead rSO2 and lower thigh rSO2. In TPP, significant changes in forehead rSO2 and lower-thigh rSO2 were not observed. Monitoring lower thigh rSO2 might be useful for preventing lower extremity pain. In BCP, forehead rSO2 decreased and lower thigh rSO2 increased from the supine position to the BCP. To prevent brain damage, anesthesiologists should pay attention to heart- and ear-level mAP.
